# Antioxidants, lysosomes and elements status during the life cycle of sea trout *Salmo trutta* m. *trutta* L.

**DOI:** 10.1038/s41598-021-85127-3

**Published:** 2021-03-10

**Authors:** Natalia Kurhaluk, Halyna Tkachenko

**Affiliations:** Department of Biology, Institute of Biology and Earth Sciences, Pomeranian University in Słupsk, Arciszewski Str. 22b, 76-200 Słupsk, Poland

**Keywords:** Biochemistry, Cell biology, Chemical biology, Developmental biology, Physiology, Zoology, Environmental sciences

## Abstract

The aim of our study was to elucidate the effects of both development stages (parr, smolt, adult, spawner), and kelt as a survival form and sex (male, female) on the functional stability of the lysosomal complex, biomarkers of oxidative stress, and element contents in the muscle tissue of the sea trout (*Salmo trutta* m. *trutta* L.) sampled in the Pomerania region (northern Poland). We have evaluated the maximal activities of lysosomal enzymes (alanyl aminopeptidase, leucyl aminopeptidase, β-N-acetylglucosaminidase, and acid phosphatase), lipid peroxidation level, and protein carbonyl derivatives as indices of muscle tissue degradation. The relationship between lysosomal activity and oxidative stress biomarkers estimated by the lipid peroxidation level and protein carbonyl derivatives was also assessed, as well as the relationships between element levels and oxidative stress biomarkers. Trends of the main effects (i.e., the development stages and sex alone, the interaction of the sex and development stage simultaneously) on oxidative stress biomarkers, lysosomal functioning, and element contents in the muscle tissue were evaluated. The study has shown sex-related relationships between the pro- and antioxidant balance and the tissue type in the adult stage as well as modifications in the lysosomal functioning induced by long-term environmental stress associated with changing the habitats from freshwater to seawater and intense migrations. The highest level of toxic products generated in oxidative reactions and oxidative modification of proteins was noted in both the spawner stage and the kelt form. The holistic model of analysis of all parameters of antioxidant defense in all development stages and sex demonstrated the following dependencies for the level of lipid peroxidation, oxidative modification of proteins, lysosomal activities, and element contents: TBARS > OMP KD > OMP AD > TAC, AcP > NAG > LAP > AAP and Cu > Fe > Ca > Mn > Zn > Mg, respectively.

## Introduction

The sea trout (*Salmo trutta* morfa *trutta* L.) is considered an extremely plastic species, which is reflected in both the diversity of life strategies implemented and the corresponding ecological forms of the species^1,2^. Sea trout are anadromous and brown trout are the resident forms of the same species *Salmo trutta* L.^3^. Any morphological and behavioral differences between them are only slight differences. In the rout, as in typical two-environmental fish, two main parts can be distinguished in the life cycle. The first part is related to the freshwater environment and the other one is associated with the sea and spawning migration. In the systematics of fish, the brown trout (*Salmo trutta*) refers to a special taxonomic unit, i.e. a morph, which is a poorly heredity-fixed modified form. This form easily returns to its original state when external conditions change accordingly. These two forms of trout (the first is a freshwater form and the other is a sea form) originate from the brown trout (*Salmo trutta*) and show diversity of their life strategies and adaptations in the evolutionary and ecological aspect^4^. Sea trout are anadromous and brown trout are the resident forms of the same species *Salmo trutta* L., and any morphological and behavioral differences between them are only slight differences^5^.

In the freshwater part of trout life, individuals adapt well to this lifestyle and never go to the sea, but exhibit lower growth intensity and fertility compared to original marine species^6^. The spawning of the eggs in the nest and their insemination initiate a long and dangerous period of transformation to which the sea trout will be exposed before returning to spawn in the river. These two forms, although derived from the same species, have acquired many specific features over a long period of development in freshwater^2^. They are different from the brown trout in the distribution range, physiology, and biochemistry associated with the processes of aquatic hydrochemistry of habitats (fresh and seawater) and not only^7^. Over the centuries, this fish species has obtained very special properties, which differentiate it from its ancestors by its hereditary morphological features. Particularly profound are the biological differences in the lifestyle, nutrition, breeding, physiological processes, and other functions directly related to the environment. In each of these two stages of the life cycle associated with the state of the environment (river, sea, river, and again the sea), the sea trout have developed a specific type of metabolism, which is the main property of any species^4,8,9^.

**Figure 1:**
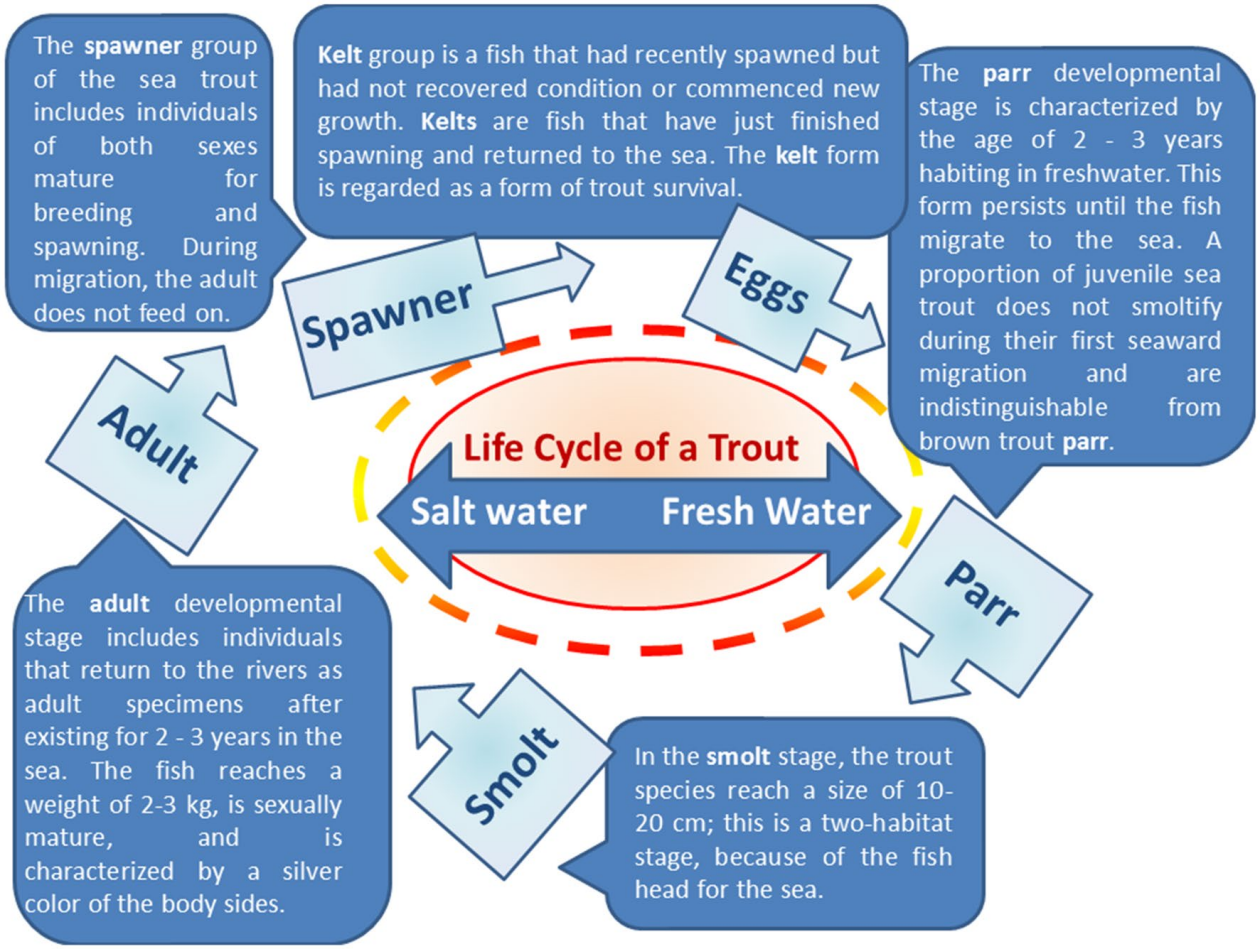
Sea trout lifecycle.

The characteristics of the main stages of the development cycle of the sea trout are shown in Fig. 1. While single eggs are extremely sensitive to environmental changes such as drops in oxygen levels and pollution, the egg batches have certain adaptive tolerance and mechanical resistance to external factors. After an incubation period of approx. four months, depending on the water temperature, the hatching begins. After 3–6 weeks from hatching, fully formed sea trout larvae begin to acquire their first food actively^10^. The parr stage is one of the key stages in the functioning of the sea trout population, as it acquires biological characteristics and behaviors that determine success in further stages of life^11^.

The growth stage of the sea trout in the river may last from one to even 7 years; however, in Poland, the migration to the sea usually starts in the second year of life, preceded by a change in body coloration during the so-called smoltification. Migration is accompanied by many physiological, morphological, and behavioral changes taking place in the fish body^2,9^, which are mainly influenced by environmental factors such as the photoperiod and temperature^12^. Changing the environmental conditions into hyperosmotic ones in relation to the content of the body fluids of the fish requires a significant energy input, which generates a high level of stress. Nevertheless, the phenomenon is extremely complex and multifactorial, and the influence of thyroid hormones on migration cannot be ruled out. Studies conducted by some authors consisted in feeding or even direct injections without taking into account all environmental and physiological factors, which did not give a complete and reliable picture of the phenomenon^13^. However, after many dramatic transformations in the organism, the trout flows into the sea as smolt. Then, rapid acceleration of growth occurs^14^.

The time and distance to which sea trout migrate depend in part on environmental conditions and genetically controlled behavior^1^. Nevertheless, it seems that the ability to adapt to the ecosystem and to find a sufficient food base for the population often determines further migrations. The life span of the sea trout in the sea is between 1 and 4 years. A signal to start the migration and spawning is the change in the length of the day, so-called photoperiods and temperature^15^. Adult sea trout fish start to turn towards the rivers in which they were born; this phenomenon is called “home instinct” or “homing”. Wild populations of the sea trout mostly reach their home streams; however, due to all types of pollution (chemical, physical), individual specimens can be caught in neighboring rivers^4,16^.

In Poland, there are two reproductive sequences in summer and autumn, in which sea trout participate, different in the terms and degree of gonad development. As the temperature drops, the coloration changes more clearly and the sex glands increase their volume many times (even up to 18% of body weight). The fish start spawning in the same year they have entered the river. Previous observations show that part of the sea trout population returns to spawn several times (even up to 10%)^16^.

Among the most informative biomarkers for assessment of the physiological state of fish, researchers have identified parameters of the intensity of lipid peroxidation as well as antioxidant enzymatic and non-enzymatic defenses, whose activity induction is a non-specific form of the body’s response to the action of stress factors^17^. These stressors are initiated by the external environment or by internal factors of neurohormonal origin in the course of fish transition from one stage of development to another^18^. Other important indicators are an oxidative modification of proteins (OMP), which are early indicators of pathological protein changers under oxidative stress^5^. At the same time, changes occurring at the molecular level are also reflected in the organism, which is manifested in a decrease in the biological characteristics of fish and changes in the values of physiological indices, which are characteristic of individuals from different areas under prolonged exposure. The intensification of these processes without the activation of compensatory mechanisms is defined as the concept of oxidative stress^19^.

The literature presents data on the chemical composition of marine and freshwater organisms; the determining factors are the species, habitat, and aquaculture conditions^20–22^. Studies were conducted on the inhabitants of both salt and freshwater areas^7^. In the water habitats, there are fast fluctuations in the concentrations of microelements and macroelements^23^. Therefore, a quantitative assessment of the content of elements in organisms in a comparative aspect (essential for anadromous fish, like salmonids) is important for practical purposes of the fish industry and for clarifying the reasons that determine the microelement composition in the basic physiological and biochemical studies of adaptive reactions^24^. However, the specific nature of the species, the type of nutrition, and the lifestyle cause differences in the content of microelements in the fish organism. There are literature data indicating the presence of individual differences in the metal content depending on the ecological specificity of fish, namely their anadromous characteristics^23,25^.

In recent years, lysosomes have been the focus of increasing attention of specialists in the biology of aquatic organisms as a target of toxic substances at the subcellular level and have been widely applied in marine pollution monitoring^26,27^. Many new properties of these cellular organelles, important for the life activity of an organism, have been revealed. It was shown that the lysosomal system was a specialized tool of cells used for realization of such important metabolic and physiological processes as catabolism of proteins, glyco- and lipoproteins, and nucleic acids, processes of accumulation, transformation, and excretion of non-native substances from the organism, receptor control, auto-, heterophagocytosis and apoptosis, adaptation and reconstruction of cell structures, and others. These studies show high functional activity, mobility, and inducibility of these cellular organelles with a big number of enzymes (up to 100) like hydrolases and oxidoreductases. Lysosomes are very sensitive to oxidative stress^28^.

Therefore, the adaptive features of oxygen-dependent processes depending on the levels of physiological elements and the activity of lysosomal enzymes for the reduction and functioning of muscle tissue in the transition from one stage of development of wild fish to another in natural environments (also considered in the age aspect) as well as sex differences are of interest to physiology, ecology, and fisheries. The literature data present research on separate elements of the functional state of muscle tissue at each stage of development of this fish species. Different effects of environmental and internal development factors are taken into account. However, there is no complete picture of interrelations and comparison of all the parameters mentioned (oxidative stress, induction of autophagic processes through lysosomal enzymes, and metal levels) in the literature.

Hence, we evaluated the impact of development stages (parr, smolt, adult, spawner), and kelt as a survival form and sex (male, female) on the functional stability of the lysosomal complex, biomarkers of oxidative stress, and element contents in muscle tissue of the sea trout (*Salmo trutta* m. *trutta* L.). We evaluated the maximal activities of lysosomal enzymes, lipid peroxidation levels, and carbonyl derivatives as indices of protein degradation in muscle tissue. The main goals of study were (i) to determine whether the activities of alanyl aminopeptidase, leucyl aminopeptidase, β-N-acetylglucosaminidase, and acid phosphatase differ between various life stages of the trout; (ii) to evaluate the relationship between lysosomal activity and oxidative stress estimated by the levels of lipid peroxidation and protein damage; (iii) to estimate the relationship between element levels and oxidative stress biomarkers; (iv) to characterize the trend of main effects (i.e. the development stage and sex) in formation of the oxidative stress biomarkers, lysosomal functioning, and element contents in the muscle tissue and the interaction of the sex and development stage effects simultaneously; (v) to evaluate important determinants of the antioxidant defenses, lysosomal stability, and metal levels from one development stage to the other.

## Materials and methods

### Statement

The study was carried out in compliance with the ARRIVE guidelines of the European Union Council and the current laws in Poland.

### Ethical approval

The study was conducted with the consent of the Bioethics Committee of Pomeranian University in Słupsk and The Marshal’s Office of the Pomeranian Voivodeship (Gdansk, Poland, License DROS.AR.MW.6052-16/10).

### Fish and experimental design

#### Characteristics of the fish experimental groups

The fish study material was sampled in the years 2008–2014 from 397 specimens of the sea trout in next developmental stages: parr (n = 113), smolts (n = 122), adults (n = 25, i.e. 13 males, 12 females), spawners (n = 113, i.e. 60 males, 53 females), and kelts (n = 24, i.e. 12 males, 12 females). Adult specimens of the sea trout were caught in the estuary of the Słupia river (Ustka city, 54° 35′ N 16° 51′ E), while the sexually mature spawners and kelts (males and females) were caught in the Słupia river (Słupsk city, Pomeranian Voivodeship, northern Poland; 54° 27′ 57″ N 17° 1′ 45″ E). In next Pomeranian rivers as Glazna, Skotawa, Kamienna, Kwacza near Słupsk city the trout specimens in the parr and smolt developmental stages were caught. Our analysis was carried out according to the sex since the phenotypic males and females possessed testicular and ovarian structures. Among the adults, spawners, and kelt forms, the sexual dimorphism is well-expressed. Fishes were collected using the electric fishing method, with the help of a power generator with a DC adapter, in close cooperation with the Landscape Park “Dolina Słupia” and the District Board of the Polish Angling Association in Słupsk.

All methods were carried out in accordance with relevant guidelines and regulations. All experimental protocols were approved by Pomeranian University in Słupsk and The Marshal’s Office of the Pomeranian Voivodeship (Gdansk, Poland, License DROS.AR.MW.6052-16/10).

Muscle tissue was taken from each fish for chemical and biochemical determinations. Tissue samples (muscle tissue) for analysis were taken and frozen in situ in liquid nitrogen, and homogenized in the laboratory of Institute of Biology and Earth Sciences, Pomeranian University in Słupsk. In order to examine the level of elements, muscle tissue samples were each taken at the dorsal fin above the lateral line.

#### Tissue homogenate preparation

The selected tissues were removed from the fish, tissue samples were excised, weighted, washed in ice-cold buffer, and minced. The trout muscle tissue samples were homogenized in ice-cold buffer contained from 100 mM Tris–HCl (pH 7.2). The minced muscle tissue was rinsed clear of blood with cold isolation buffer and homogenized on ice. Homogenates were centrifuged at 3000 × *g* for 15 min at 4 °C. After centrifugation, the supernatant was collected and frozen at − 20 °C until analyzed. Protein contents were determined with the Bradford method^29^ with bovine serum albumin as a standard in duplicate.

#### Tissue isolation for lysosomal enzymes assays

The minced tissues were rinsed with cold isolation buffer contains 0.15 M KCL to remove the blood. Tissues homogenized in a glass Potter–Elvehjem homogenizer with a motor-driven Teflon pestle on ice. The isolation buffer consisted of 0.25 M sucrose and 2 mM EDTA (pH 7.0). The homogenates of several muscles 20% (wt/vol) were prepared to next differential centrifugation according to the method described by authors^30^. After centrifugation the supernatant fractions were saved and used after resuspendation in 50 mM acetic acid/sodium acetate buffer, pH 5.0 in two freeze–thaw cycles.

### Biochemical assays

#### Thiobarbituric acid reactive substances (TBARS) assay

The intensity of lipid peroxidation processes was determined by the measurement of thiobarbituric acid reactive substances (TBARS) according to method^31^ and optical density was measured at a wavelength of 540 nm.

#### Protein carbonyl derivatives assay

The level of oxidative modified proteins (OMP) was evaluated by the content of protein carbonyl derivatives (ketone-2,4-dinitro-phenylhydrazone) in the reaction with 2,4-dinitro-phenylhydrazine (DNFH) by method of authors^32^ and optical density was measured at a wavelength of 370 nm with the molar extinction coefficient 22,000 M^−1^ cm^−1^.

#### Total antioxidant capacity assay

The total antioxidant activity was determined by the inhibition of Fe^2+^/ascorbate-induced oxidation of Tween 80 with the next determination of TBARS level at the 532 nm by method in work^33^. Absorbance of blank solution without sample was accepted as 100%. The level of TAC in sample (%) was calculated according to the absorbance of blank.

#### Lysosomal enzyme assays

The activity of alanyl aminopeptidase (EC 3.4.11.2.) and leucyl aminopeptidase (EC 3.4.11.1.) was determined spectrophotometrically according to authors^34^. Reaction was initiated by 50 μl of sample and 500 μl of substrate incubation media with DMF (Serva, Germany), 60 min incubation at 37 °C, pH 6.0, next, the 500 μl of stop buffer consisting of Fast Blue BB Salt dissolved in 2% Tween 20 (Sigma, USA) was added. Absorbance was measurement at 540 nm. l-alanyl-2-naphtylamine in 0.1 M PBS buffer was used as a substrate for alanyl aminopeptidase activity determination and l-leucyl-2-naphtylamine in 0.1 M PBS pH 7.0 buffer was used as a substrate for leucyl aminopeptidase activity determination.

The activities of other lysosomal enzymes such as acid phosphatase (EC 3.1.3.2.), β-N-acetylglucosaminidase (EC 3.2.1.30.) were determined spectrophotometrically as 4-nitrophenyl derivatives at 420 nm as described by method proposed authors^35^. The activities of enzymes were expressed in nM/h and in mg protein accordingly method^29^.

### Determination of elements concentration

In order to determine the concentration of Mg, Ca, Zn, Mn, Cu and Fe in muscle tissue, they were subjected to mineralization in a mixture of nitric acid (HNO_3_) and hydrogen peroxide H_2_O_2_. The determination of micro- and macroelements content was made by flame atomic absorption spectrophotometry (air-acetylene flame) method using the Analyst 300 spectrometer. The elements Mg, Ca, Zn, Mn, Cu and Fe were determined using the following wavelengths: 285.2, 422.7, 213.9, 279.5, 324.8 nm and 248.3 nm respectively. During calcium and magnesium level value determine in purpose to eliminate the effect of phosphorus, a lanthanum chloride La^3+^ solution was added to all samples, which would provide 0.5% of the concentration of La^3+^ in the tested solutions. The results of the content of Mn, Cu, Zn, Fe are expressed in mg per kg dry weight, whereas the results of the content of Ca and Mg are expressed in mg per 100 g dry weight.

### Statistical analysis

The results of analysis were expressed as mean ± S.D. Normal distribution were tested using the Kolmogorov–Smirnov test (p > 0.05) and the homogeneity of variance was assessed using Levene’s test. Statistical analysis was carried out in a double way: biomarkers of oxidative stress, lysosomal enzymes activity and elements levels were compared with those in each developmental stage of the sea trout, while metabolism biomarkers, lysosomes and metals were analyzed separately. The combined effect of sex and developmental stages, as well as its significance (main effects), was compared with the data of the oxidative stress, lysosomal parameters and elements separately. Differences were considered significant at p < 0.05. The significance of differences in the parameters' value, and between all studied groups was determined using one-way analysis of variance (ANOVA) and multifactorial analysis of variance (MANOVA). For unequal observations, Tukey’s post-test was used.

A multivariate analysis of variance (MANOVA) for two fish stages (adults, spawners) and the kelt form we have using to verify the hypothesis of the impact of the sex and developmental stages on the antioxidant defense, lysosomes stability and elements value in the muscle tissue. The use of multivariate significance tests of the main effects (sex, development stages, and its combined effects) allowed to obtain statistically significant relationships for all three values. In our fish model approach, to combine the impact of two factors dependently of sea trout development (sex and the stages), we adopted a two-way classification model in the form, where X_ijk_—the value of the dependent variable, µ—mean, α_i_—main effect of the sex factor, β_j_—main effect of the development stage factor; (αβ)_ij_—the effect of the interaction of the sex and development stage factors; ε_ijk_—random experimental error.

The correlation and regression analysis comprised the correlation coefficient (r), regression equation, and significance of these dependencies (P). We used the coefficients of multiple correlation analysis (R), the coefficient of determination (R^2^), and its corrected form reduced by random errors (R^2^ adjusted) in the data analysis for the description of the full model. We used the SS test to describe the share of all analyzed data of oxidative stress, lysosomal parameters and elements content for assessment data with the F test and its significance. All statistical calculations were performed on separate data from each individual with STATISTICA 13.3 software (StatSoft Inc., Poland); www.statsoft.pl/Site_License/slJ3876apwmp.php, serial number: JPZ805I387617AR-R.

## Results

### Functioning of pro/antioxidant balance and oxidative stress

The analysis of the level of one of the final lipoperoxidation products, i.e. the MDA content, was shown to have a stable increasing tendency with the age of fish. It should be noted that, during the selected development stages, the level of lipoperoxidation processes in the muscle tissue differed significantly [F_7,364_ = 94.39, p = 0.000] with age and transition to every next stage of fish development. This can also be seen in the minimum and maximum values obtained as well as the median level (Table 1). We observed a statistically significant increase in the level of lipoperoxidation processes in the final stages of the biological development of this fish species, namely the spawner and kelt form, which did not differ between the sexes.

**Table 1:** TBARS products (nmol MDA mg^−1^ protein) in the muscle tissue of sea trout (*Salmo trutta* m. *trutta* L.) during different developmental stages.

Development stage	Mean value, nmol MDA mg^−1^ protein	Minimum, nmol MDA mg^−1^ protein	Maximum, nmol MDA mg^−1^ protein	Median, nmol MDA mg^−1^ protein
Parr	42.31 ± 16.59	14.09	91.17	39.47
Smolt	52.21 ± 19.34 a	10.23	88.37	51.02
Adult male	37.50 ± 8.05	24.94	48.58	39.27
Adult female	50.98 ± 10.44	29.72	67.83	53.15
Spawner male	88.35 ± 7.45 bb, c, d	76.32	100.95	88.80
Spawner female	87.99 ± 6.76 bb, c, dd	71.08	106.31	87.94
Kelt male	97.53 ± 4.69 cc, e, ee	84.67	101.19	99.54
Kelt female	93.95 ± 6.22 e, cc, dd	83.64	101.24	93.75

**Figure 2:**
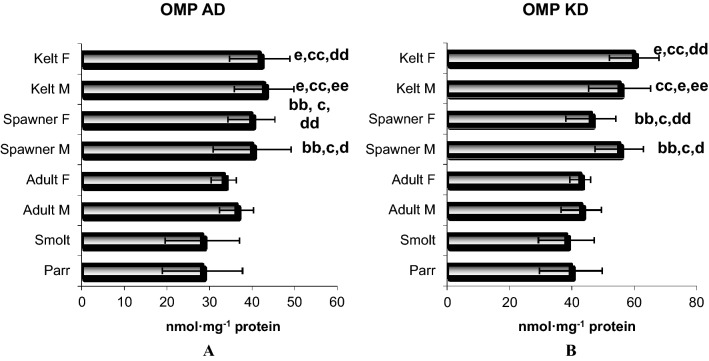
Aldehydic derivatives (AD) (nmol/mg protein) and ketonic derivatives (KD) of oxidatively modified proteins (OMP) (nmol/mg protein) content in the muscle tissue of the sea trout (*Salmo trutta* m. *trutta* L.) during different development stages. Results are expressed as mean ± S.D. Differences between experimental groups were analysed by one-way ANOVA and Tukey’s post-hock test for unequal observations. Differences were considered significant at p < 0.05. Legend: developmental stages—Parr (n = 113), Smolt (n = 122), Adult Male (n = 13) and Adult Female (n = 12), Spawner Male (n = 33) and Spawner Female (n = 55), Kelt male (n = 12) and Kelt Female (n = 12). Significant differences between groups are designated as follows: a—Smolt group vs. Parr group; aa—Adult Male or Adult Female vs. Parr group; b—Adult Male or Adult Female vs. Smolt group; bb—Spawner Male group or Spawner Female group vs. Parr group; c—Spawner Male group or Spawner Female group vs. Smolt group; cc—Kelt Male group or Kelt Female group vs. Smolt group; d—Spawner Male group vs. Adult Male group; dd—Spawner Female group vs. Adult Female group; e—Kelt Male group or Kelt Female group vs. Parr group; ee—Kelt Male group vs. Adult Male; f—Kelt Female group vs. Adult Female group; ff—Kelt Male group vs. Spawner Male group; j—Kelt Female group vs. Spawner Female group.

The action of active oxygen forms leads to violation of the native conformation of proteins with the formation of large protein aggregates or fragmentation of a protein molecule (modified protein level, OMP). Lipid radicals can also cause fragmentation of protein molecules. Carbonyl derivatives have become one of the most frequently used markers of oxidative stress, which was analyzed in the next part of our study on the development-induced changes in the level of protein carbonyl derivatives OMP AD and OMP KD. Our results show a significant effect of the development stage on the increase in these parameters. The data of OMP AD [F_7,364_ = 72.56, p = 0.000] and OMP KD [F_7,364_ = 69.33, p = 0.000] different significantly with age. The results are shown in Fig. 2. However, we demonstrated a statistically significant increase in OMP AD and OMP KD in the muscle tissue accompanying the transition to each stage of fish development and the increasing age.

**Figure 3:**
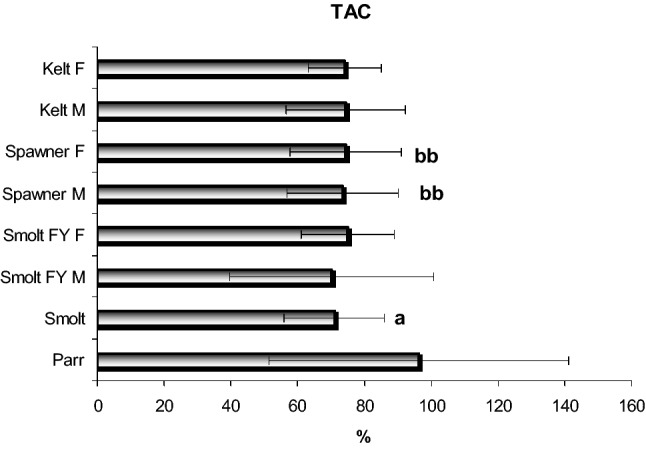
Total antioxidant capacity (%) in the muscle tissue of the sea trout (*Salmo trutta* m. *trutta* L.) during different developmental stages. Results are expressed as mean ± S.D. Differences between experimental groups were analysed by one-way ANOVA and Tukey’s post-hock test for unequal observations. Differences were considered significant at p < 0.05. For Legend—see Fig. 2.

The cellular mechanisms of resistance to oxidative stress are studied in a holistic system using the total antioxidant capacity parameter. These results are presented in Fig. 3. Our results indicate significant dependencies [F_7,364_ = 5.56, p = 0.001] in the TAC level in the muscle tissue. We observed a decrease in the TAC parameter value already in the second stage of development as a smolt and in adult fish in comparison with the parr stage.

### Lysosome enzymes

Normally, the composition and condition of membranes and enzymes are modified not only by the free radical and lipid peroxidation processes but also by membrane-bound, free (solubilized), and lysosomal enzymes. Under the influence of various factors, lysosomal activity or its cytoplasm content may increase (in particular, due to the development of acidosis, which increases the yield of lysosome enzymes and their subsequent activation). The study of the trend in the lysosomal enzyme activity in the muscle tissue at the different stages of development was the next stage of our study.

Our results showed (Table 2) that the alanyl aminopeptidase (AAP) activity [F_7,364_ = 34.86, p = 0.000] changed statistically significantly during the development stages as follows: there was a significant increase in the smolt stage with subsequent reduction in its activity in the adult male compared to the parr stage and an important increase in the kelt (male, female) form.

**Table 2:** Alanyl aminopeptidase, leucyl aminopeptidase, acid phosphatase, and β-N-acetylglucosaminidase (nM/h/mg protein) activities in the muscle tissue of the sea trout (*Salmo trutta* m. *trutta* L.) during different developmental stages.

Development stage	AAP, nM/h/mg protein	LAP, nM/h/mg protein	AcP, nM/h/mg protein	NAG, nM/h/mg protein
Parr	321.31 ± 46.59	214.09 ± 21.25	191.17 ± 19.25	139.47 ± 19.58
Smolt	452.21 ± 47.34 a	210.23 ± 35.21	88.37 ± 5.47 a	151.02 ± 12.45
Adult male	237.50 ± 38.05 b	224.94 ± 33.14	248.58 ± 36.41 b	239.27 ± 41.33 b
Adult female	350.68 ± 10.44 b	229.72 ± 44.11	267.83 ± 52.10 b	253.15 ± 14.78 b
Spawner male	388.65 ± 57.45	376.32 ± 28.66 bb, c, dd	200.95 ± 25.41 c	388.80 ± 25.11 c
Spawner female	487.79 ± 76.74	371.08 ± 77.12 bb, c, dd	206.31 ± 18.74 c	387.94 ± 52.07 bb, c, dd
Kelt male	697.53 ± 84.69 cc, e, ee	484.67 ± 25.99 cc, e, ee	301.19 ± 21.33 cc, e, ee	399.54 ± 47.52 cc, e, ee
Kelt female	693.95 ± 86.22 e, cc, dd	483.64 ± 85.21 e, cc	301.24 ± 51.07 e, cc	393.75 ± 31.22 e, cc

*AAP* alanyl aminopeptidase, *LAP* leucyl aminopeptidase, *AcP* acid phosphatase, *NAG* β-N-acetylglucosaminidase.

See as Table 1.

In our research, the leucyl aminopeptidase (LAP) activity [F_7,364_ = 25.16, p = 0.000] was higher in the spawner (male, female) stage and kelt (male, female) form. In the first stage of the sea trout growth (parr, smolt, and adult), we did not find any significant changes in the activities of LAP.

In the present study, we observed statistically significant dependencies in the acid phosphatase (AcP) activity [F_7,364_ = 121.86, p = 0.000] in the sea trout muscle tissue during the analyzed development stages. In the second stage of trout growth (smolt), the activity of the AcP enzyme was lower than in the first stage (parr); however, we noticed an increasing trend in the activity of this enzyme from the parr stage. A significant increase in the activity was observed in the kelt fish stage of development.

Similar tendencies were observed for the β-N-acetylglucosaminidase (NAG) activity [F_7,364_ = 16.86, p = 0.000] in the sea trout muscle tissue during the development stages. It should be noted that the functioning of the pro/antioxidant balance is associated with significant intensification of lipoperoxidation, oxidative modification of proteins, and the associated significant intensification of lysosomal enzyme activity depending on the development stages and the sex of individuals.

### Metal level

In the present study, we observed statistically significant changes in the concentration of calcium [F_7,364_ = 125.36, p = 0.000] and magnesium [F_7,364_ = 117.92, p = 0.000] depending on the development stage (Table 3). The highest level of calcium was observed in the smolt development stage with a subsequent distinct tendency towards a statistically significant decrease in adult female and spawner male individuals. It was observed that the highest magnesium level in the parr and smolt development stages decreased statistically significantly in the male and female adult stages. Irrespective of the sex, the spawner and kelt forms were characterized by a reduced magnesium level in the muscle tissue, and the value was lower than in the parr and smolt stages of fish development.

**Table 3:** Calcium and Magnesium levels (mg 100 g^−1^ dry weight) in the muscle tissue of the sea trout (*Salmo trutta* m.*trutta L.*) during different developmental stages.

Development stage/elements	Ca, mg 100 g^−1^ dry weight			Mg, mg 100 g^−1^ dry weight		
	Mean value	Minimum	Maximum	Mean value	Minimum	Maximum
Parr	8.68 ± 3.72	2.47	29.65	29.40 ± 14.35	8.58	87.32
Smolt	11.21 ± 6.25 a	2.96	37.67	29.02 ± 15.04	5.02	95.08
Adult male	4.65 ± 1.67 aa	1.98	6.76	8.88 ± 4.83 aa, b	1.53	17.91
Adult female	2.73 ± 0.68 aa	1.73	3.81	8.04 ± 2.23 aa, b	2.35	9.92
Spawner male	3.67 ± 1.36 bb, c, d	1.55	7.36	12.93 ± 7.33 bb, c	2.49	32.39
Spawner female	5.71 ± 2.32 bb, c, dd	1.76	11.01	13.61 ± 4.72 bb, c	3.83	22.04
Kelt male	5.99 ± 2.09 ff	3.33	10.44	12.25 ± 4.22 e, cc	7.10	19.09
Kelt female	5.48 ± 2.25	3.81	10.44	12.15 ± 3.74 e, cc	7.92	19.09

See as Table 1.

The results of the concentration of such metals as iron, copper, manganese, and zinc are presented in Table 4. The highest level of Cu, Fe, Mn, and Zn was observed for the spawner male, parr, spawner male, and parr stages, respectively. This tendency in the determined metal levels was in line with the stages of development, with the lowest content in the trout muscle tissue presented in the following way adult male for Cu and Fe, adult male and female for Mn, and spawner male for Zn.

**Table 4:** Selected elements levels (mg kg^−1^ dry weight) in the muscle tissue of the sea trout (*Salmo trutta* m. *trutta* L.) during different developmental stages.

Development stage/elements	Cu, mg kg^−1^ dry weight	Fe, mg kg^−1^ dry weight	Mn, mg kg^−1^ dry weight	Zn, mg kg^−1^ dry weight
Parr	0.74 ± 0.38	8.08 ± 5.41	0.031 ±	5.58 ± 2.72
Smolt	0.63 ± 0.32	4.33 ± 3.54 a	0.03 ± 0.01	6.11 ± 3.16
Adult male	0.40 ± 0.09	4.13 ± 1.75	0.02 ± 0.01	4.12 ± 1.47
Adult female	0.41 ± 0.09	3.91 ± 1.07	0.02 ± 0.01	4.65 ± 2.13
Spawner male	0.91 ± 0.45 d	6.66 ± 4.55	0.05 ± 0.02 bb, c	3.22 ± 1.98 bb, c
Spawner female	0.60 ± 0.27	7.06 ± 3.58 c	0.04 ± 0.01 bb, c, dd	5.47 ± 2.28
Kelt male	0.47 ± 0.17	7.08 ± 3.70	0.03 ± 0.01	5.66 ± 3.21
Kelt female	0.68 ± 0.16	5.07 ± 0.82	0.04 ± 0.01	5.05 ± 1.67

See as Table 1.

### Sex and development stage relationship

To verify the hypothesis of the impact of the sex and development stage on in the lipid peroxidation level, lysosomal enzyme degradation processes, and concentration of selected elements, we decided to compare these tendencies using a multivariate analysis of variance. We used this type of significant dependencies for three sea fish forms, where the sexual dimorphism was reflected in the adult, spawner, and kelt forms. As shown by the present statistical analysis, the main effect was only noted for the interaction of the development stage and a combination of the development stage and sex factors. The results are presented in Table 5. Similar results were obtained for the data on the element level. The determination of the significance of the main effects of the interaction between the sex and developmental stage allow a conclusion that these factors and their combination have a particular impact on the activity of lysosomal enzymes.

**Table 5:** Multivariate significance tests and effective hypothesis decomposition for three stages (adults, spawner, and kelt) for oxidative stress biomarkers parameters, lysosomal enzymes and elements of the muscle tissue of the sea trout.

Main effects	Test value	F	p
**Oxidative stress**			
Sex	0.947	1.796	0.134
Development stage	6.6393	214.118	0.000
Sex × development stage	0.1950	6.288	0.000
**Lysosomal enzymes**			
Sex	0.947	6.786	0.000
Development stage	4.739	108.118	0.000
Sex × development stage	0.147	5.295	0.000
**Elements**			
Sex	0.934	1.481	0.190
Development stage	0.398	8.43	0.000
Sex × development stage	0.503	9.188	0.000

For analysis of these three independent processes in the holistic model, we used the multiple correlation coefficient (R), the coefficient of determination (R^2^), and adjusted R^2^ (Table 6). The dependencies of lipid peroxidation processes, oxidatively modified protein profiles, and total antioxidant capacity value can be presented as follows: TBARS > OMP KD > OMP AD > TAC. The dependencies on the case of important lysosomal enzymes are as follows: AcP > NAG > LAP > AAP. The research has shown the following relationships between the selected elements in the sea trout muscle tissue: Cu > Fe > Ca > Mn > Zn** > **Mg.

**Table 6:** SS test of oxidative stress parameters for a full model of the oxidative biomarkers data, lysosomal enzymes and elements in the muscle tissue and SS for residues for the three stages of sea trout development (first-year smolt, spawner, and kelt).

Parameters	Multiple R	Multiple R^2^	Multiple adjusted R^2^	F	p
TBARS	0.931	0.867	0.862	171.43	0.000
OMP AD	0.348	0.121	0.088	3.62	0.004
OMP KD	0.607	0.368	0.344	15.28	0.000
TAC	0.073	0.005	0.033	0.14	0.983
AAP	0.412	0.170	0.132	7.12	0.000
LAP	0.478	0.228	0.201	9.14	0.000
AcP	0.785	0.616	0.521	32.11	0.000
NAG	0.558	0.311	0.269	9.54	0.000
Ca	0.497	0.247	0.218	8.57	0.000
Cu	0.522	0.273	0.245	9.82	0.000
Fe	0.522	0.273	0.245	3.18	0.009
Mn	0.470	0.221	0.191	7.43	0.000
Mg	0.342	0.117	0.083	3.47	0.005
Zn	0.403	0.162	0.130	5.08	0.000

In the present study, we observed statistically significant dependencies between the parameters of lipoperoxidation processes in muscle tissue and the sea trout development stage estimated as TBARS products, the level of oxidatively modified proteins and TAC, lysosomal enzyme activity, and the level of elements using correlation and regression analysis (Table 7). These correlations varied in the case of each of the development stages and depended significantly on the sex of fish in stages characterized by clear sexual dimorphism.

**Table 7:** Correlation intergroup interdependencies between the parameters of oxidative stress, lysosomes enzymes and elements in the muscle during different development stages of the sea trout.

Developmental stages	Significant relationships, r, p < 0.05	Developmental stages	Significant relationships, r, p < 0.05
Parr	TBARS-OMP AD 0.20	Spawner male	Cu–Fe 0.56
	OMP AD-Fe 0.22		Cu–Mn 0.71
	OMP AD-Mn 0.33		Cu–Mg 0.76
	OMP KD-Fe − 0.24		OMP AD-AcP 089
	OMP KD-Mn 0.42		OMP KD-NAG 087
	Mn–Cu 0.64		
Smolt	OMP AD-Zn − 0.39	Spawner female	TAC-Ca − 0.34
	OMP KD-Zn − 0.35		TAC-Zn − 0.32
	TAC-Fe 0.23		Fe–Cu 0.56
	TAC-Zn − 0.23		Ca–Mg 0.45
	Ca–Cu 0.34		Ca–Zn 0.59
			TBARS-LAP 0.88
Adult male	Zn–Mg 0.80	Kelt male	TBARS-OMP AD − 0.67
	Zn–Fe 0.75		TBARS-Ca − 0.58
	Zn–Mn 0.84		TBARS-Mg − 0.59
	TBARS-AAP 0.76		OMP KD-Ca 0.72
			Cu–Zn 0.79
			OMP KD-AAP 089
Adult female	TBARS-OMP AD − 0.87	Kelt female	OMP KD − Fe 0.70
	Ca–Cu 0.72		Ca–Cu − 0.84
	Cu–Mn 0.93		Zn–Mg 0.94
	Mg–Fe 0.87		TBARS-LAP 0.88
	TBARS-LAP 0.58		OMP KD-AcP 082

## Discussion

The present study demonstrated the impact of sex, development stages, and their combination on the muscle tissue in the wild sea trout *Salmo trutta* m. *trutta* L. Previously, we estimated similar effects in the liver tissue^5^. However, in comparison with liver tissue, muscle tissue is characterized by other physiological and biochemical characteristics associated with the structure of myofibril and energy loads. This makes it important for the realization of strategic evolutionary goals of fish associated with long-distance movements, which is especially important for the anadromous type of life^36^.

Compared with other classes of vertebrates, muscle tissue of fish has its own characteristics^37,38^. Their trunk muscles consist of a series of myomeres, separated from each other by interlayers of connective tissue (myosepts). Depending on the ratio of organelles, the muscle fibers are divided into white (light), red (dark), and intermediate. Although the processes of biochemical analysis of fish muscle tissue under the influence of factors of different genesis at different stages of development or as elements of aquaculture are sufficiently represented in the literature^37,38^, we have not found reports similar to our results. Our data allow us to analyze a complete model of interconnected processes of oxidative stress generation, which in turn causes destructive induction of lysosomal autophagy, and the content of elements at each stage of wild trout development. Analysis of changes in oxidative stress markers is a very important part of analysis of fish adaptive abilities and condition. Therefore, our research consisted in determining the level of oxidative stress markers, e.g. aldehyde and carbonyl groups of oxidation-modified proteins and malondialdehyde levels as indicators of the functional abilities of fish.

There are several important findings in the study. Firstly, the statistically significant dependencies of our multivariate statistical analysis in the holistic model showed the leading role of lipoperoxidation processes in the analysis of oxidative stress biomarkers, namely, 86.2% of the whole analyzed model, as well as high values of correlations. This is related to the stable tendency in muscle tissue towards an increase in the intensity of lipoperoxidation processes during the transition from one stage to another in this species of wild fish, with a significant increase in the level of these processes in both males and females at the final stages of development as the spawner and kelt form.

The chemical composition of tissues of hydrobionts, especially the skeletal muscles of fish, is not stable and can undergo significant changes, affecting the nature of oxygen diffusion and the nature of oxygen-dependent processes. First of all, this concerns the content of lipids and water, which can determine the intensification of oxidative stress processes. It is known that the oxygen solubility in lipids is 4 times higher than in the cytoplasm. There is a natural dynamics of the lipid content and, accordingly, the intensity of their peroxidation, associated with annual cycles (spawning, feeding, and migration) as shown by various authors^39^. Hormonal regulation contributes significantly to the adaptation of this species to changing environmental conditions. It is known that one of the most significant hormone functions (shown for thyroid hormones by author^40^) is the adaptation of the individual to the habitat. Against the background of fish adaptation to the biotope is the process of their sexual maturation, which is regulated by many factors, including sex steroid hormones. In favorable habitat conditions associated with rapid growth, the trout may reach puberty already at the age of 1+ and 2+.

Free radical oxidation processes are normally carried out continuously in all tissues and cells of living organisms. Peroxide oxidation of lipids proceeds as a chain of exothermic chemical processes of oxidative modification of neutral lipids and phospholipids. Free radical reactions, supported by special regulatory systems at a low steady-state level, participate in normal metabolic processes and regulatory functions of the cell. The increase or decrease in the process of lipid peroxidation alters the composition of cell membranes, their structural organization, and the functional activity of the cell.

The study of the level of oxidatively changed proteins, which we conducted at the different stages of trout development, provides important information about the degree of damage to protein structures. Oxidative changes in proteins are an inherent effect of aerobic cellular metabolism. The formation and accumulation of oxidized protein products depends on the rate of production of reactive oxidizing agents (with disrupted activity of antioxidant systems), on protein sensitivity to oxidation, and on the degradation rate of oxidized proteins. The important role of iron in these processes was also confirmed by the results of our statistical analysis of the correlations between oxidative stress parameters and iron at the subsequent stages of trout development: OMP KD-Fe r =  − 0.24 and OMP AD-Fe r = 0.22 for the parr stage, TAC-Fe r = 0.23 for the smolt stage, and OMP KD-Fe r = 0.70 for the kelt female stage.

The action of active oxygen forms leads to violation of the native conformation of proteins with the formation of large protein aggregates or fragmentation of a protein molecule. The hydroxyl radical most often causes protein aggregation and, in combination with the superoxide anion, fragmentation accompanied by formation of low-molecular fragments. Lipid radicals can also cause fragmentation of protein molecules. The mechanism of aggregate formation is as follows: under the action of oxidants, the native conformation of a number of protein domains is disturbed. As a result, the number of hydrophobic residues on the globular surface increases, which causes the formation of large protein conglomerates.

Secondly, the increase in the level of the modified proteins in our study was associated with the increased lipoperoxidation and activity of lysosomal enzymes. The correlations between the level of the lipoperoxidation process, estimated through TBARS products and OMB, were as follows: TBARS-OMP AD r =  − 0.87 for the adult female stage and TBARS-OMP AD r =  − 0.67 for the kelt male stage. As can be seen from the results of the above essential statistical analysis of correlations, we obtained these dependencies mainly for the final stages of trout growth, which are accompanied by elevated lipoperoxidation levels and modification of proteins. The accumulation of oxidized protein products leads to such damage as fragmentation, modification of amino acid residues, and aggregation^19^. These processes may lead to loss of the biological function of the protein. Oxidative protein modifications result from oxidative stress, which can occur in cells via disorders in physiological processes or as a result of negative external factors.

Lipid peroxidation products are cytotoxic, mutagenic, and carcinogenic^41^. These compounds modify the physical properties of cell membranes, increasing membrane permeability to H+ ions and other polar substances^26^. Activation of hydrolases (lysosomal, membrane-bound, and free) is one of the stages of stress action. Some authors have proposed that the autophagic removal of oxidatively damaged organelles and proteins provides a second tier of defense against oxidative stress^28^. It has also been proposed that organisms making up functional ecological assemblages in fluctuating environments, where up-regulation of autophagy should provide a selective advantage, may be pre-selected to be tolerant of induced oxidative stress^42^.

Normally, the composition and condition of membranes and enzymes are modified not only by free-radical and lipoperoxide processes, but also by membrane-bound, free (solubilized), and lysosomal enzymes. The lysosomal-autophagic system appears to be a common target for many environmental areas, as lysosomes accumulate many toxic metals and organic xenobiotics, which perturb normal function and damage the lysosomal membrane. Importantly, autophagic reactions frequently involving reduced lysosomal membrane integrity or stability appear to be effective generic indicators of cellular well-being in aquatic organisms^27,28^. Under the influence of various factors, their activity or content in the hyaloplasm of cells may increase (in particular, due to the development of acidosis, which increases the yield of enzymes from lysosomes and their subsequent activation). Glycerophospholipids and membrane proteins as well as cell enzymes undergo intensive hydrolysis. This is accompanied by a significant increase in membrane permeability and a decrease in the kinetic properties of the enzymes. As we have shown in the holistic model, acidic phosphatase plays a leading role in initiating lysosomal destruction processes (52.1% in the whole model of the selected lysosomal enzymes). This is also confirmed by the results of the correlation analysis showing the relationship between these processes (OMP AD-AcP r = 0.89 for the parr stage and OMP KD-AcP r = 0.82 for the kelt female stage). We have established relationships (Table 7) between the processes of lysosomal activity and lipoperoxidation in the following trout stages: adult male TBARS-AAP r = 0.76, adult female TBARS-LAP r = 0.58, OMP KD-NAG r = 087 for the spawner male stage, and TBARS-LAP r = 0.88 for the spawner female stage.

It is known that, unlike other vertebrates, the muscle tissue of fish regularly undergoes periodic destruction, since it is mainly due to muscle tissue that the fish organism replenishes protein deficiency in any stressful situations^43^. Lysosomes have been shown to take part in these processes. However, in fish that live in normal ecological conditions, as soon as start feeding, the muscle fiber structure is restored with the restoration of the number of muscle proteins, i.e. the process of degeneration is replaced by the process of intracellular physiological regeneration. Thus, the ability of fish to utilize muscle proteins with their subsequent recovery is a morphological adaptation that has developed in evolution and has been established at the genetic level.

Thirdly, the Cu, Fe, Ca, Mn, Zn**,** and Mg concentration in the fish muscle, depending on the stage of development and sex, was associated with development of a number of adaptations^7^. As shown by our data, there was a significant change in the lipid peroxidation level and function of lysosomal enzymes, depending on the environment (fresh or salt water). A certain oxygen deficiency can be experienced by salmonid fish during the transition from fresh water bodies to the marine waters, which is associated with a change in the habitat, restructuring of the water-electrolyte composition of the external homeostasis environment, and energy supply systems for the restructuring of these processes. It should be noted that the content of minerals in fish depends on many others factors, such as the species, age, physiological status, habitat, nutrient content of the water body, salinity and temperature of the water^7^, season, or harvest time^23,25^. However, no less important are the reciprocal interactions between metals at each stage of development and their combined effect (stage of development and sex). Our research has shown the leading role of copper and iron in the induction of lipoperoxidation and lysosomal destruction processes. These statistically significant patterns of reciprocal muscle tissue interrelations were observed for the following stages: Cu–Mn r = 0.64 parr, Cu–Ca r = 0.34 smolt, between Cu and Fe and Mn and Mg for spawner male and female and kelt male and female. It should be noted that magnesium in the general analysis of the statistical model of the main effects was assigned an insignificant role, but there were similar dependencies of magnesium and other metals in almost all stages. It is undoubtedly important that lipid peroxidation also results in inhibition of membrane enzymes and transporting proteins, e.g. (Ca^2+^, Mg^2+^-ATP-ases), as shown by^44^. Ultimately, peroxidation reactions can result in impaired integrity of intracellular membranes and the plasma membrane^41^. Lipid peroxidation also results in inhibition of membrane enzymes and transporting proteins, Ca^2+^, Mg^2+^-ATP-ases.) Ultimately, peroxidation reactions can result in impaired integrity of intracellular membranes and plasma membrane.

The real necessities for future study and importance of these research on this economically valuable species of migratory fish consists in the search for ecophysiological relationships between different developmental stages of sea trout (e.g. parr, smolts, sexually mature spawners and spawning kelts) developing in marine and freshwater environments and the degree of bioaccumulation of metals in response to environmental stress. The research we are conducting, in addition to providing a factual background, will enable conservation measures to further increase the numbers of this valuable fish species in Polish rivers.

## Conclusions

The functioning of the pro/antioxidant balance in the muscle tissue reflects the course of individual developmental stages of the trout and age. The results regarding the destructive activation of lysosomal mechanisms, such as alanyl aminopeptidase, leucyl aminopeptidase, acid phosphatase, and β-N-acetylglucosaminidase enzymes, show a compensatory effect depending on the developmental stages and the sex of individuals. The research has shown sex-related relationships between the oxidative stress data during adult stage as well as modifications of the lysosomal functioning induced by long-term environmental stress associated with changing the habitats from freshwater to salty water and intense migrations. The highest level of toxic oxidation reaction products and oxidative modification of proteins was noted in the spawner stage and kelt form. The holistic model for the analysis of all parameters of antioxidant protection in all developmental stages and sexes demonstrated the following dependencies for the level of oxidative stress, lysosomal activities, and element contents: TBARS > OMP KD > OMP AD > TAC, AcP > NAG > LAP > AAP, and Cu > Fe > Ca > Mn > Zn** > **Mg respectively.
